# Nanotechnology against human cytomegalovirus in vitro: polyanionic carbosilane dendrimers as antiviral agents

**DOI:** 10.1186/s12951-021-00809-4

**Published:** 2021-03-03

**Authors:** I. Relaño-Rodríguez, M. S. Espinar-Buitrago, V. Martín-Cañadilla, R. Gómez-Ramirez, J. L. Jiménez, M. A. Muñoz-Fernández

**Affiliations:** 1 Section Head Immunology, Laboratorio InmunoBiología Molecular, Hospital General Universitario Gregorio Marañón (HGUGM), Madrid, Spain; 2 Instituto de Investigación Sanitaria Gregorio Marañón (IiSGM), Madrid, Spain; 3 Departamento de Química Orgánica y Química Inorgánica, Universidad de Alcalá, Instituto de Investigación Química “Andrés M. del Río” (IQAR), UAH, Alcalá de Henares, 28871 Spain; 4 Spanish HIV-HGM BioBank, Madrid, Spain; 5 Networking Research Center on Bioengineering, Biomaterials and Nanomedicine (CIBER-BBN), Madrid, Spain

**Keywords:** Polyanionic carbosilane dendrimers, HCMV, Ganciclovir

## Abstract

**Background:**

Human cytomegalovirus (HCMV) is a worldwide infection, causing different troublesome in immunosupressed patients and very related to Human Immunodeficiency Virus 1 (HIV-1) infection, mainly in developing countries, with a co-infection rate of 80% in Africa. The high cost of present treatments and the lack of routinely tests in these countries urge the necessity to develop new molecules or strategies against HCMV. The new treatments should be low-cost and capable of avoiding the emerging problem of resistant virus. Nanoparticles play an important role in several viral infections. Our main focus is to study the potential activity of polyanionic carbosilane dendrimers (PDC), which are hyperbranched molecules with several sulfonate or sulfate groups in their periphery, against different viruses.

**Results:**

We studied the activity of G1-S4, G2-S16 and G2-S24P PDCs in MRC-5 cell line against HCMV infection by several plaque reduction assays. Our results show that dendrimers present good biocompatibility at the concentrations tested (1–50 µM) for 6 days in cell culture. Interestingly, both G2-S16 and G2-S24P showed a remarked inhibition at 10 µM against HCMV infection. Results on attachment and virucidal assays indicated that the inhibition was not directed to the virus or the virus-cell attachment. However, results of time of addition, showed a longer lasting activity of these dendrimers in comparison to ganciclovir, and the combination of G2-S16 or G2-S24P with ganciclovir increases the HCMV inhibition around 90 %.

**Conclusions:**

Nanotechnology, in particular polyanionic carbosilane dendrimers, have proved their potential application against HCMV, being capable of inhibiting the infection by themselves or enhancing the activity of ganciclovir, the actual treatment. These compounds represent a low-cost approach to fight HCMV infections.
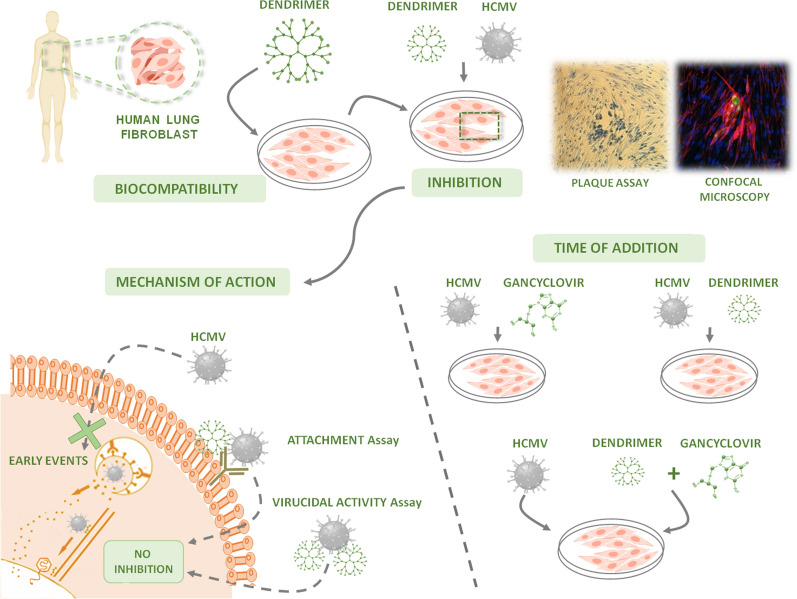

## Introduction

The human herpesvirus 5 or human cytomegalovirus (HCMV) is a widespread pathogen, belonging to betaherpesviridae family. The HCMV genome consists of a double stranded DNA with approximately 230,000 bp. The genome is enclosed by an icosahedral capsid (100–110 nm diameter) and the mature viral particle has a diameter of 150–200 nm [1].

The HCMV infects and replicates in a wide variety of cells, including epithelial cells of gland and mucosal tissue, smooth muscle cells, fibroblasts, macrophages, dendritic cells, hepatocytes and vascular endothelial cells [2]. This broad cell tropism causes systemic spread in the human body, and inter-host spread.

In immunocompetent individuals, HCMV infection is asymptomatic in most cases or yield minor symptoms. However, asymptomatic individuals are able to spread the HCMV by several body fluids [1]. Foremost, in immunosupressed individuals, as AIDS patients, elders or transplant recipients, HCMV infection can lead to dissemination and life-threatening end-organ diseases [3]. Moreover, congenital HCMV (cCMV) infections can cause severe clinical deficiencies in the development of the foetus and can produce an impact of 0.7 in newborns, being the pathogen of congenital transmission more common in the worldwide [4]. Congenital infections are usually caused by a primary infection of the mother during pregnancy with an intrauterine transmission rate of 40–50%.

The HCMV represents an important co-infection in HIV-1 infected patients. HCMV is associated with increased morbidity and mortality [5], specifically in African population, where the seroprevalence of HCMV infection is near 80% in HIV-1 patients. Data obtained from patients in the South Africa indicate that in severely HCMV patients, ganciclovir therapy could be life-saving. However, in this country, the access to common treatments is not always available as a consequence of their high cost. Moreover, no routinely tests for HCMV infection are performed in most developing countries [6]. Due to these factors, the discovery of new treatments is essential, since new resistances are emerging in current treatments [7–9].

Our group has focused on the study of different polyanionic carbosilane dendrimers (PCDs). These dendrimers have shown their ability to prevent or even to eliminate the transmission of several sexual infectious diseases such as HIV-1, Human Herpes Virus (HSV-1), (HSV-2) or Hepatitis C Virus (HCV) [10–14]. In last decades the nanotechnology has achieved many goals in different fields, from basic application to biomedicine, emerging new applications and treatments [15–18]. Dendrimers are hyperbranched nanoparticles, consisting of a silicon or polyphenolic core, surrounded by tree-like branches in which generations are defined by the number of repeated layers of the branches units. In the periphery of branches, several functional groups are located, which can be modified, allowing dendrimers to develop several functionalities.

Different groups have shown that the probability of HCMV infection increases in HIV-1 infected patients due to different protein and cofactor interactions, and vice versa [18–20]. In those cases, a low-cost treatment capable of inhibiting one, or even both infections, could drastically reduce the great incidence of both diseases in different developing countries. Altogether, along with the high prevalence of HCMV and HIV-1 co-infection in several countries, the developing of a cost-effective treatment that would be capable of diminishing both infections is of remarked importance. In our work, we present G2-S16 and G2-S24P PCDs, which are capable of reducing HCMV infection showing good biocompatibility. Although the mechanism of action of G2-S16 or G2-S24P dendrimer against HCMV infection has not been deciphered yet, the promising results, along with the cost-effective production of these nanocompounds, position those PCDs as good antiviral agents against HCMV.

Our objective is to demonstrate that nanoparticles, specifically PCDs with sulfonate or sulfate groups in their periphery, are capable of inhibiting HCMV infection *in vitro.*

## Materials and methods

### Dendrimers

PCDs G1-S4 with silicon core and 4 sulfate groups in periphery, G2-S16 with silicon core and 16 sulfonate groups in the periphery, and G2-S24P with a polyphenolic core and 24 sulfonate groups in the periphery. All dendrimers ranged between 1 and 20 nm being bigger as the generation of the dendrimers increases. PCDs were synthesized and analyzed according to methods reported by the Dendrimers for Biomedical Applications Group of University of Alcalá (Madrid, Spain) [21–23]. NMR spectroscopy data confirmed the identity of compounds: G1-S4 1H NMR (DMSO-d6): 7.85 (s, 4H, NCHCN), 4.26 (s, 8H, SiCH2CH2CH2CH2N), 3.91 (s, 8H, CCH2CH2OSO32), 2.85 (s, 8H, CCH2CH2OSO32), 1.77 (s, 8H, SiCH2CH2CH2CH2N), 1.25 (s, 16H, SiCH2CH2), 0.50 (s, 24H, SiCH2), 20.10 (s, 24H,Si(CH3)2). 13 C{1H} NMR (DMSO-d6): 143.1 (NCHCN), 121.8 (NCHCN), 64.3 (CCH2CH2OSO32), 48.3 (SiCH2CH2CH2CH2N), 33.1 (SiCH2CH2CH2CH2N), 25.5 (CCH2CH2OSO32) 19.9–13.8 (SiCH2CH2 y SiCH2), 23.8 (Si(CH3)2). 29Si NMR (DMSO-d6): 1.7 (Si(CH3)2) G2-S16 (1H-NMR (D2O): − 0.21 (br. s, 12 H, SiMe), − 0.16 (s, 48 H, SiMe2), 0.28 (m, 16 H, SiCH2CH2CH2N), 0.43 (m, 48 H, SiCH2CH2CH2Si), 1.21–1.31 (m, 40 H, SiCH2CH2CH2Si and SiCH2CH2CH2N), 2.28 (m, 16 H, CH2CHCH2N), 2.76 (m, 32 H, NCH2CH2SO3), 2.86 (m, 32 H, NCH2CH2SO3). 13 C-NMR{1H} (D2O): − 4.4 (SiMe), − 3.5 (SiMe2), 12.4 (SiCH2CH2CH2N), 17.7–18.5 (CH2), 20.1 (CH2), 46.7 (NCH2CH2SO3), 48.0 (NCH2CH2SO3), 56.9 (CH2CH2CH2N). 29Si-NMR (D2O): G0-Si not observed, 1.1 (G1-Si), 2.1 (G2-Si)) and G2-S24P 1H-NMR (D2O): − 0.18 (br. s, 99 H, SiMe), 0.28 (m, 30 H, SiCH2), 0.41 (m, 72 H, SiCH2), 1.05–1.20 (m, 66H, CH2CH2CH2), 1.60 (m, 2 H, OCH2CH2), 2.27 (m, 24 H, CH2CHCH2N), 2.75 (m, 48 H, NCH2CH2SO3), 2.85 (m, 48 H, NCH2CH2SO3), 3.42 (m, 2 H, OCH2), 5.64 (br. s, 3 H, C6H3).

**Fig. 1:**
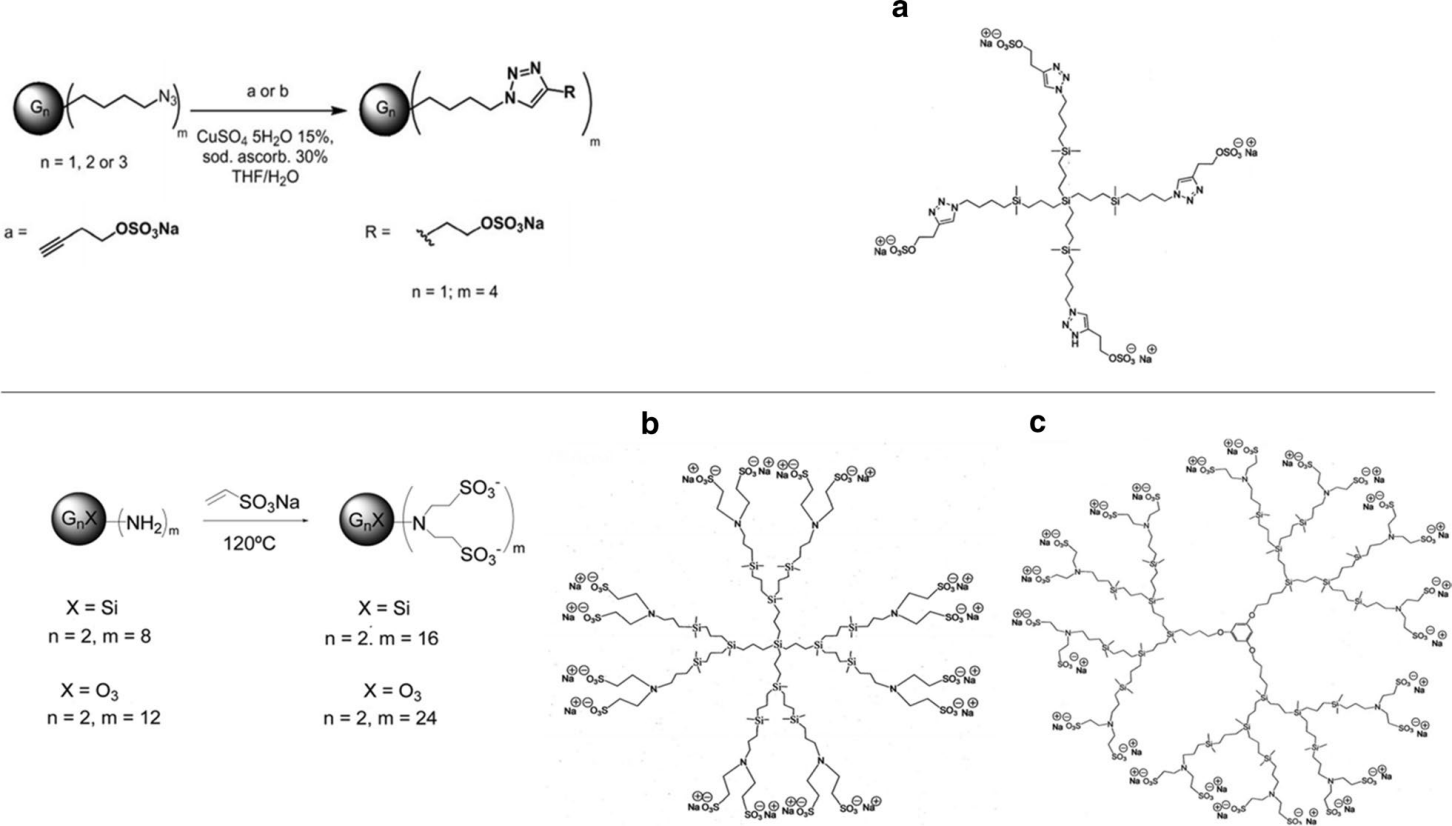
Schematic synthesis route and molecular representation of dendrimers. **a** G1-S4 with silicon core and 4 sulfate end groups, **b** G2-S16 with silicon core and 16 sulfonate end groups and **c** G2-S24P with polyphenolic core and 24 sulfonate end groups were represented. The generation of dendrimers is determined by considering that each generation corresponds to the number of repeating layers of silicon atoms

13 C-NMR{1H} (D2O): − 4.6 (SiMe),−3.4 (SiMe2), 12.7 (SiCH2CH2CH2N), 18.5 (CH2), 20.0 (CH2), 20.7 (CH2), 22.4 (CH2), 47.1 (NCH2CH2SO3), 47.8 (NCH2CH2SO3), 57.2 (CH2CH2CH2N), 66.9 (OCH2), 93.6 (C6H3, CH), 160.6 (i-C6H3). 15 N-NMR (D2O): − 343.2. 29Si-NMR (D2O): 1.7 (only one peak was observed) (Additional file 1). Stock solution of dendrimers (5 mM) and subsequent dilutions to work concentrations were prepared in nuclease-free water (Promega, Madrid, Spain). The schematic structures of PDCs are presented in Fig. 1. NMR spectroscopy data NMR spectra were recorded on a Varian Unity VXR-300 (300.13 (1H), 75.47 (13 C) MHz) or on a Bruker AV400 (400.13 (1H), 100.60 (13 C), 79.49 (29Si) MHz).

### Cells and viruses

Human lung fibroblast cells (MRC-5) (ATCC CCL-171) were cultured in Dulbeco’s Modified Essential Medium (DMEM) supplemented with 10% Heat- inactivated Fetal Bovine Serum (FBS) and 1% antibiotics cocktail containing 125 mg/ml ampicilin, 125 mg/ml cloxaciclin and 40 mg/ml gentamicin (Normon, Madrid, Spain). The viral strain HCMV_AD−169_ (ATCC VR-538) was expanded and titrated in MRC-5 cell line by plaque assay with serial dilutions. Stock aliquots at 3.5 × 10^6^ PFU/mL were prepared by ultracentrifugation and stored at − 80 ºC.

### Mitochondrial activity assay

The mitochondrial toxicity of G1-S4, G2-S16 and G2-S24P PCDs was tested by the 3-(4-5-dimethylthiazol-2-yl)-2,5-diphenyltetrazolium bromide (MTT) assay (Sigma, St. Louis, USA) according to manufacturer's instructions. Briefly, 1 × 10^5^ cells/well of MRC-5 cells were seeded in 96-well plates and treated with the desired compounds for 6 days in a concentration range (1–50 µM). After incubation, culture medium was discarded and 220 µl of a 1:11 MTT (5 mg/ml)/OptiMEM solution was added to cultured MRC-5 cells. After 3 h, the supernatant was removed, and formazan crystals were dissolved in 200 µl DMSO (Sigma, St. Louis, MO, USA) absorbance was read in a Berthold Plate Reader at 570 nm. All points were performed in triplicate. DMSO 10 % was used as death cellular control. Non-treated cells were used as viability control.

### Membrane integrity assay

Cellular toxicity was measured by the lactate deshidrogenase (LDH) assay CytoTox 96® Non-Radioactive Cytotoxicity (Promega, Spain, Madrid) following manufacturer’s instructions. Briefly, 1 × 10^5^ MRC-5 cells were seeded in 96-well plates and treated with the desired compounds for 6 days in a concentration range (1–50 µM). After the incubation period, MRC-5 cells were lysed in 0.9 % Triton X-100 (Promega, Spain, Madrid) for 45 min at 37 ºC and 50 µl of LDH reagent (Promega, Spain, Madrid) was added for 30 min at room temperature, protected from light. Absorbance was read in a Berthold Plate Reader at 490 nm. All points were performed by triplicate. Non-treated MRC-5 cells were used as viability control.

### Inhibition assay

The potential activity of PCDs against HCMV_AD−169_ infection was measured by plaque reduction assays. Briefly, 7.5 × 10^4^ cells/well of MRC-5 cells were seeded in 24-well plates and incubated at 37 ºC for 24 h. After incubation, MRC-5 cells were treated with G1-S4, G2-S16 or G2-S24P for 1 h at 37 ºC and infected with 60 PFU of HCMV_AD−169_ for 2 h at 37 ºC, inoculum was discarded and DMEM supplemented with 2 % FBS was added. MRC-5 cells were incubated for 6 days at 37 ºC, MRC-5 cells need 6 days after the virus was added to develop cytopathic effects by HCMV. After that supernatant was discarded and MRC-5 cells were dyed with Methylene Blue (Sigma, St. Louis, MO, USA) for plaque counting. Percentage of inhibition is given as the reduction of the plaques formed with dendrimers treatments in comparison with the infection control.

### Attachment assay

To study if the inhibition of PCDs is directed to block the virus-membrane interaction, membrane attachment studies were performed by plaque reduction assays. Briefly, 7.5 × 10^4^ MRC-5 cells/well were seeded in 24-well plates and incubated at 37 ºC for 24 h. After incubation, MRC-5 cells were pre-cooled at 4 ºC for 15 min and treated with G1-S4, G2-S16 or G2-S24P for 1 h at 4 ºC and infected with 60 PFU of HCMV_AD−169_ for 2 h at 4 ºC, inoculum was discarded and DMEM supplemented with 2 % FBS was added. MRC-5 cells were incubated for 6 days at 37 ºC, supernatant was discarded and MRC-5 cells were dyed with Methylene Blue for plaque counting. Percentage of inhibition is given as the reduction of the plaques formed with dendrimers treatments with regard to the infection control.

### Virucidal activity

To study the direct interaction between dendrimers and virus, virucidal activity by plaque reduction assays were performed. Briefly, 7.5 × 10^4^ MRC-5 cells/well were seeded in 24-well plates and incubated at 37 ºC for 24 h. We combine G2-S16 or G2-S24P at 10 µM with 60 PFU/ml of MRC-5 cell free HCMV_AD−169_ for 2 h at 37 ºC. After incubation, the eppendorfs were centrifugated at 12,000 rpm for 1 h at 4 ºC, supernatant was discarded, rinsed with PBS and centrifugated at 12,000 rpm for 1 h at 4 ºC to eliminate the compound. Finally, supernatant was replaced with fresh DMEM supplemented with 2% FBS and added to MRC-5 cells. Triton X-100 at 0.1% was used as positive control of disrupting HCMV_AD−169_ membrane. HCMV_AD−169_ infection was revealed as described before.

### Time of addition

To specify the inhibition check point of dendrimers in the viral cycle, the activity of G2-S16 and G2-S24P dendrimers was studied at different time points post-infection by plaque reduction assays. Briefly, 7.5 × 10^4^ MRC-5 cells/well were seeded in 24-well plates and incubated at 37 ºC for 24 h. After incubation, MRC-5 cells were infected with 60 PFU of HCMV_AD−169_ and treated at 0, 24, 48, 72 and 96 h post-infection, either with G2-S16, G2-S24P, ganciclovir or a combination of PCDs with ganciclovir. In all conditions, MRC-5 cells were incubated for 6 days after infection at 37 ºC, and HCMV_AD−169_ infection was revealed as described before. Percentage of inhibition is given as the reduction of the plaques formed with G2-S16 dendrimer, ganciclovir or G2-S16/ganciclovir treatments with regard to the infection control.

### Confocal microscopy

To confirm the results obtained by plaque reduction assays, we performed confocal microscopy in MRC-5 cells. Briefly, 7.5 × 10^4^ MRC-5 cells were plated and incubated for 24 h in 24-well plates, in which was previously added a 12 mm cover-glass per well pre-treated with Collagen IV (Sigma-Aldrich, St. Louis, MO, USA) for 1 h. After incubation, MRC-5 cells were treated with G2-S16 for 1 h at 37 ºC and infected with 60 PFU of HCMV_AD− 169_ for 2 h at 37 ºC, inoculum was discarded and fresh DMEM, supplemented with 10 % FBS, was added. MRC-5 cells were incubated for 6 days, then supernatant was discarded and cells were fixed in 4 % paraformaldehide (PFA; Panreac, Barcelona, Spain) for 10 min, washed 3 times in PBS and permeabilized with 0.1 % Triton X-100 (Sigma-Aldrich, St. Louis, MO, USA) for 10 min. After incubation MRC-5 cells were washed 3 times in PBS, blocked with 1 % bovine serum albumin (BSA Sigma-Aldrich, St. Louis, MO, USA) and 0.1 % Triton X-100 in PBS for 30 min and incubated with Cytomegalovirus pp65 Antibody, ALEXA FLUOR® 488 Conjugated (bioNova cientifica S.L) and phalloidin ALEXA FLUOR® 555 Conjugated antibody for 1 h, MRC-5 cells were washed 3 times in PBS and dyed with DAPI (Sigma-Aldrich, St. Louis, MO, USA) 10 min. Finally, the cover glasses were mounted in slides and analyzed in a Leica TSC SPE confocal microscope (Leica Microsystems, Wetzlar, Germany). Fluorescence was analyzed using ImageJ software (National Institute of Health, USA).

### Statistics

Statistical analysis was performed using GraphPad software Prism v.5.0 (GraphPad Software, San Diego, CA USA) and results were assessed by using a paired *t*-test between two groups (control versus different dosages of compounds or treated versus ganciclovir and G2-S16 or G2-S24P) (*p < 0.05; **p < 0.005; ***p < 0.001). Time of addition assays were analyzed by a 2 ways ANOVA between 2 groups (ganciclovir versus combinations with G2-S16 or G2-S24P).

## Result and discussion

### Biocompatibility of dendrimers in human lung fibroblast cell line

**Fig. 2:**
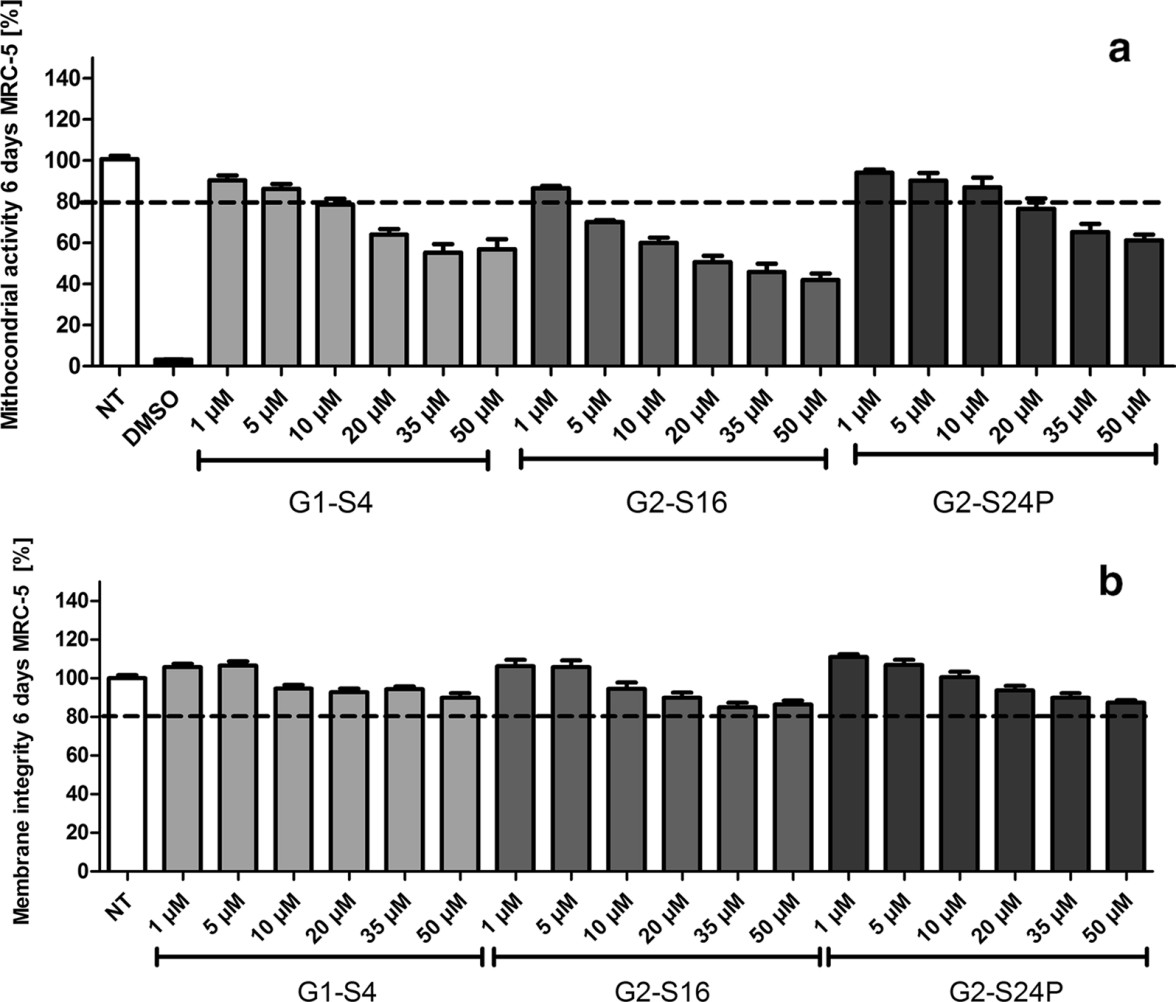
Biocompatibility studies of polyanionic carbosilane dendrimers (PCDs). **a** MTT and **b** LDH assays were performed in MRC-5 cells for 6 days treatments. MRC-5 cells were treated with increased concentration range (1 µM – 50 µM) of G1-S4, G2-S16 or G2-S24P PCD. Non-treated (NT) MRC-5 cells were used as cell viability control. DMSO was used as cell death control. NT, non-treated control; DMSO, Dimethyl sulfoxide. The mean values (mean ± SD) of at least three independent experiments are shown (*p < 0.05; **p < 0.005; ***p < 0.001)

The analysis of biocompatibility of G1-S4 dendrimer with 4 sulfate groups in periphery, G2-S16 dendrimer with 16 sulfonate groups in periphery and G2-S24P dendrimer with a polyphenolic core and 24 sulfonate groups in periphery was performed by studying the mitochondrial activity (MTT) and cell membrane integrity (LDH) of treatments at increasing concentrations (1, 5, 10, 20, 35, 50 µM) for 6 days in MRC-5 cell culture (Fig. 2). The MTT results in MRC-5 cells show that G1-S4, G2-S16 and G2-S24P PCDs at more than 1 µM cause a dose-dependent reduction in mitochondrial activity. In contrast with MTT results, that shows a specific state of mitochondrial activity and is not categorical of cell death [24]. LDH results, which refers to cell membrane integrity, show different results, indicating that treatments with G1-S4, G2-S16 and G2-S24P PCDs for 6 days do not cause any damage in MRC-5 cells membranes, even the tendency was similar.

### G1-S4, G2-S16 and G2-S24P PCDs inhibition against HCMV_AD−169_ infection

**Fig. 3:**
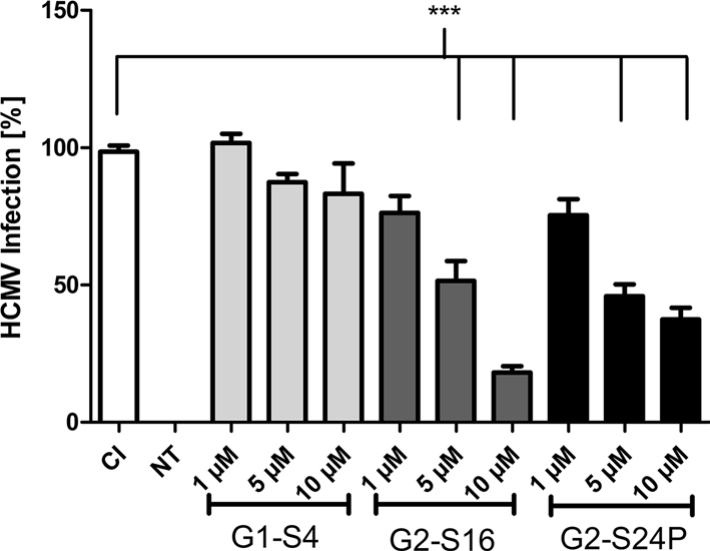
Inhibition activity of dendrimers against HCMV_AD−169_. Plaque reduction assays were performed in MRC-5 cells for 6 days treatments. MRC-5 cells were pre-treated for 1 h at 37 ºC with increased concentrations of G1-S4, G2-S16 and G2-S24P PCDs (1, 5 and 10 µM) and infected with 60 PFU at 37 ºC. Plaques were counted after 6 days with a methylene blue dye. Percentage of infection is represented as the number of plaques in treatments in regard of the control of infection. CI, Control of Infection;  NT, non-treated control. The mean values (mean ± SD) of at least four independent experiments are shown (*p < 0.05; **p < 0.005; ***p < 0.001)

Previously published reports of PCDs against HIV-1 [25], HSV-2 [11, 12] or even HCV [10] propose PCDs as alternative agents against several viral infections [26]. The studies of the potential activity of G1-S4, G2-S16 and G2-S24P PCDs against HCMV_AD−169_ infection was performed by plaque reduction assays in MRC-5 cells (Fig. 3). The results represent the percentage of infection in regard of the infection control. Results show that G1-S4 dendrimer at all concentrations tested do not cause any reduction in the number of plaques. In contrast G2-S16 and G2-S24P dendrimers show a significant reduction of the HCMV_AD−169_ infection in comparison with the infection control. Both second generation dendrimers at 5 µM cause approximately 50% of inhibition, reaching the maximum inhibition for G2-S24P. However, G2-S16 dendrimer at a concentration of 10 µM leads to 80% of plaque reduction in MRC-5 cells, showing a dose-dependent mechanism of action.

### Confocal microscopy of HCMV_AD−169_ infection in MRC-5 cell line

In order to confirm the inhibitory activity of G2-S16 against HCMV_AD_-169, previously observed by plaque reduction assays in the MRC-5 cell line, confocal microscopy images were taken of treatments with G2-S16 dendrimer.

**Fig. 4:**
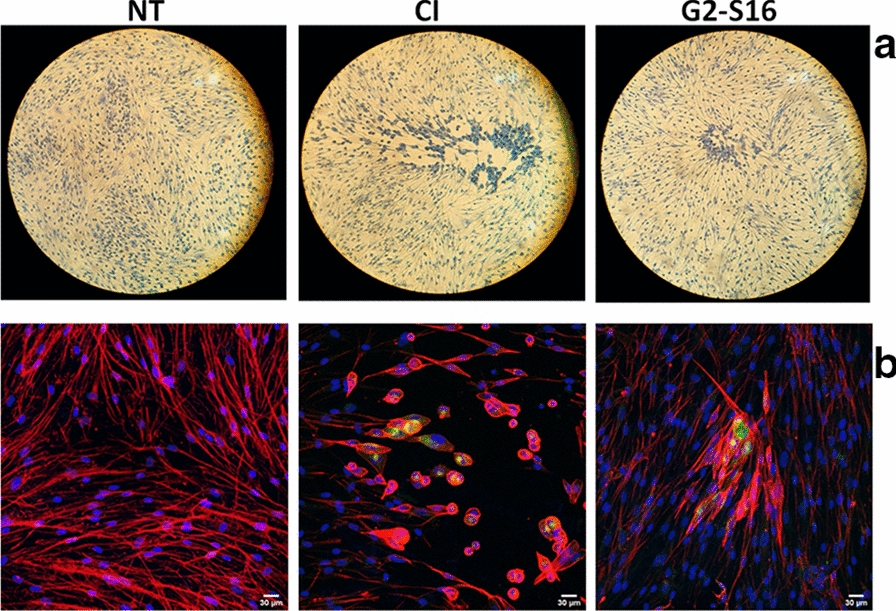
Representative images of HCMV_AD**-**169_ plaques in treatments with G2-S16 dendrimer. **a** Optical and **b** confocal representative images of MRC-5 cells. Briefly, MRC-5 cells were plated and treated with G2-S16 dendrimer (10 µM) for 1 h and infected with 60 PFU for 2 h at 37 ºC. After 6 days incubation **a** MRC-5 cells were dyed with methylene blue and images were taken on a DMIL Leica microscope at 4X augment. **b** MRC-5 cells were treated with HCMVpp65 ALEXA FLUOR® 488 Antibody Conjugated for viral particles visualization, phalloidin ALEXA FLUOR® 555 Conjugated and DAPI for membrane and nucleus localization. NT: non-treated; CI: control of infection

The fibroblast-like morphology of MRC-5 cells was visualized by interaction with actin filaments, through a red phalloidin stain, HCMV_AD_-169 infection was visualized thanks to the labelling with an antibody against the viral protein integument pp65 in green colour and cell nuclei were marked in blue by DAPI staining. The results indicate that treatments with G2-S16 dendrimer, compared to control of infection, not only reduce notably the number of plaques as shown in Fig. 4, but also the size of the plaques itself decreases in about 40 % (*data not shown*). We hypothesize that this decreasing in the plaque size is probably meaning, either that dendrimer is not allowing a normal replication of the virus or that the new transcribed virus is not able to infect the nearest cells, as we can observe viral protein in the centre of the plaque with G2-S16 dendrimer treatment, but not the same spreading as control of HCMV_AD_-169 infection.

### Inhibition of virus‐cell membrane attachment

**Fig. 5:**
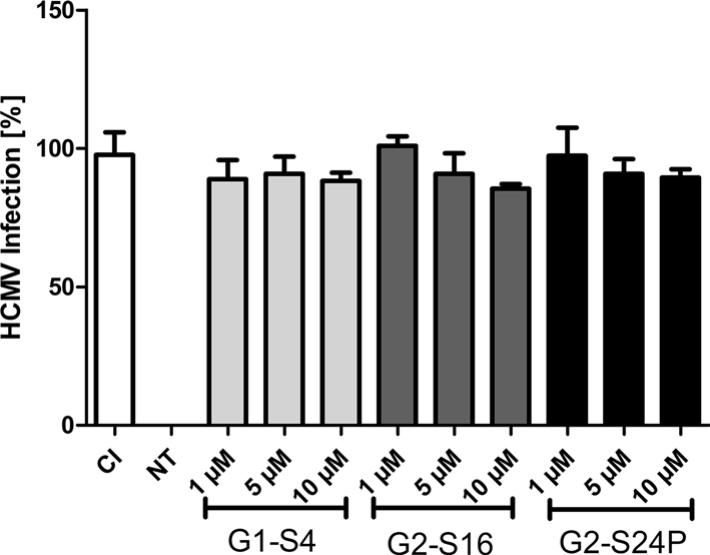
Activity of PCDs at cell membrane-virus attachment. Plaque reduction assays were performed in MRC-5 cells for 6 days treatments. MRC-5 cells were pre-cooled at 4 ºC and pre-treated for 1 h at 4 ºC with increased concentrations of G1-S4, G2-S16 and G2-S24P PCDs (1, 5 and 10 µM) and infected with 60 PFU at 4 ºC. Plaques were counted after 6 days with a methylene blue dye. Percentage of infection is represented as the number of plaques in treatments in regard of the control of HCMV_AD−169_ infection. CI: Control of Infection NT = non-treated control. The mean values (mean ± SD) of at least four independent experiments are shown (*p < 0.05; **p < 0.005; ***p < 0.001)

To study if the mechanism of action of dendrimers against HCMV_AD-169_ infection was directed to block the interaction of the virus at the cell membrane, attachment assays were performed. In order to observe this interaction, the cell membrane was forced at 4 ºC to be less permeable either to virus and dendrimer. Previously data published in our group with those PCDs against HSV-1 and HSV-2 infection, indicated that G1-S4 and G2-S16 dendrimers inhibition mechanism was directed to inhibit virus-cell membrane interaction [10]. In contrast, in our results (Fig. 5) HCMV_AD-169_ infection was not reduced in this assay at any PCDs concentration, leading to the hypothesis that in this case, G1-S4, G2-S16 and G2-S24P PCDs are not inhibiting the HCMV_AD−169_ entry to the MRC-5 cells. There is no inhibition at the cell membrane level. In fact, these results indicate that the inhibition observed in previous assays is occurring in an intracellular level, and that PCDs do not have an unspecific mechanism of action, even more, the behaviour depends on the viral cycle and the host cell.

### Virucidal activity of G2-S16 and G2-S24P dendrimers against HCMV_AD-169_

**Fig. 6:**
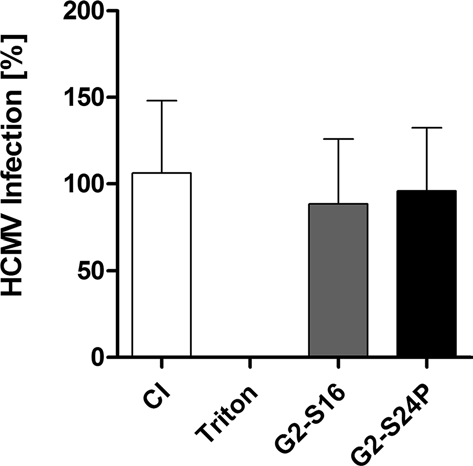
Virucidal activity of G2-S16 or G2-S24P dendrimers against HCMV_AD−169_. Plaque reduction assays were performed in MRC-5 cells for 6 days treatments. G2-S16 or G2-S24P dendrimer at 10 µM was incubated with 60 PFU for 2 h at 37 ºC. By several centrifugations at 12,000 RPM virus was pelleted, resuspended in fresh DMEM and added to MRC-5 cells for plaque reduction assays. Plaques were counted after 6 days with a methylene blue dye. Percentage of HCMV_AD−169_ infection is represented as the number of plaques in treatments in regard of the control of infection. Triton X-100 was used as a positive control of virus membrane disruption. CI: Control of Infection, NT: non-treated control. The mean values (mean ± SD) of at least four independent experiments are shown (*p < 0.05; **p < 0.005; ***p < 0.001)

Antiviral activity of PCDs has already been described, specifically direct interaction of G2-S16 dendrimer against the viral glycoprotein 120 (gp120) in HIV-1 infection [13], leading to a disruption on viral membrane, reducing the viral infection. To study the direct interaction between G1-S4, G2-S16 and G2-S24P PCDs against cell-free HCMV_AD−169_, virucidal assays were performed. The results (Fig. 6) show that direct contact of PCDs with HCMV_AD−169_ do not cause any disruption on virus membrane, leading to a complete infection in G2-S16 and G2-S24P dendrimers treatments in comparison with Triton X-100.

### Time‐of‐addition of G2-S16 and G2-S24P dendrimers during HCMV_AD−169_ infection

**Fig. 7:**
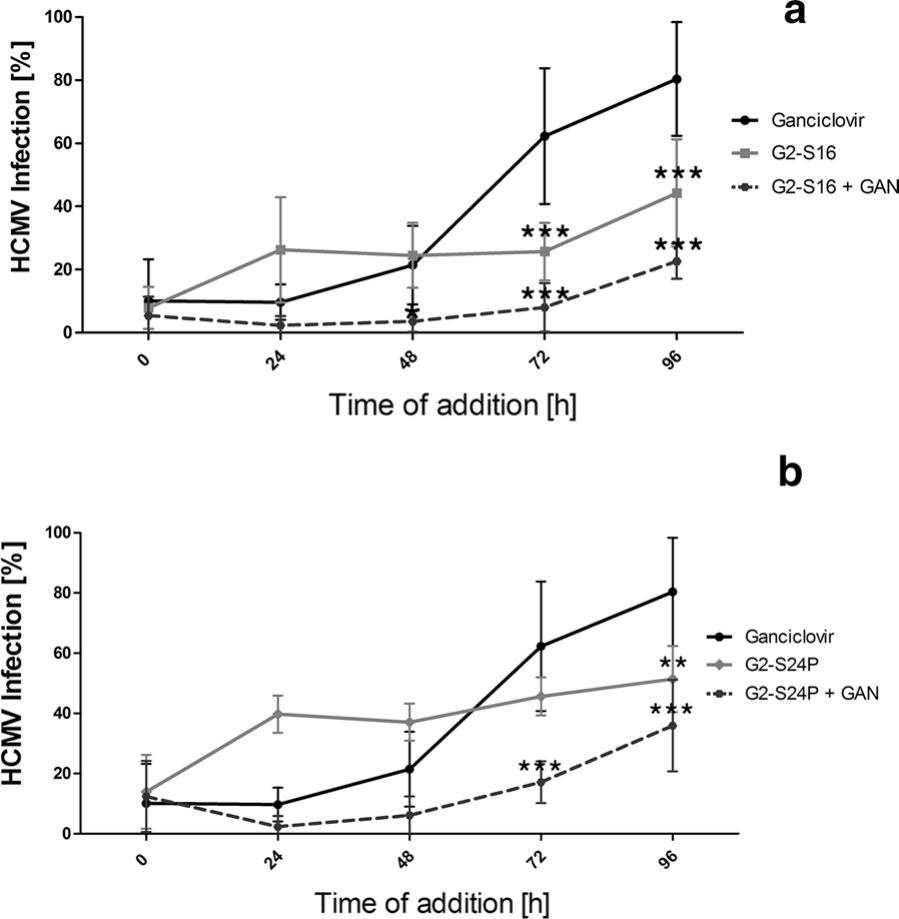
Time of addition of G2-S16 or G2-S24P dendrimer and their combinations with ganciclovir. Plaque reduction assays were performed in MRC-5 cells for 6 days. MRC-5 cells were seeded, infected with 60 PFU and treated at different time points (0, 24, 48, 72 and 96 h post-infection) with either **a** ganciclovir, G2-S16 dendrimer and G2-S16 dendrimer + ganciclovir combination **b** ganciclovir, G2-S24P dendrimer, and G2-S24P + ganciclovir. Plaques were counted after 6 days with a methylene blue dye. Percentage of HCMV_AD−169_ infection is represented as the number of plaques in treatments in regard of the control of HCMV_AD−169_ infection. CI: Control of infection, NT: non-treated control. The mean values (mean ± SD) of at least four independent experiments are shown (*p < 0.05; **p < 0.005; ***p < 0.001)

A time of addition assay was performed to study the mode of action of G2-S16 and G2-S24P dendrimers either alone, or in combination with ganciclovir during a viral infection cycle in MRC-5 cells (Fig. 7). Ganciclovir results, measured by plaque reduction assay, show that it maintains its inhibitory activity if added 48 h post HCMV_AD−169_ infection. After that time point, the HCMV_AD−169_ inhibition is reduced to 40% at 72 h and 20% at 96 h. However, we obtained different results with dendrimers single treatment, in the case of G2-S16, inhibition activity is maintained longer than we observed with ganciclovir. During the first stages of the HCMV_AD−169_ cycle, G2-S16 at 10 µM is able to maintain 70–80% of HCMV_AD−169_ inhibition activity, interestingly at the 48 h time point, ganciclovir and G2-S16 reach the same inhibition rate, but in contrast of the election drug, G2-S16 inhibition activity last longer, reaching 55–60 % of HCMV_AD−169_ inhibition even if treated 96 h post-infection. We also performed a combination of G2-S16 dendrimer and ganciclovir and we observe that the plaque number at almost all time points is greatly reduced, specifically, for 72 h post infection all points show an HCMV_AD−169_ inhibition of almost 90–95% of the infection, and this combination reduces its inhibitory activity at 96 h, with almost 80% of HCMV_AD−169_ inhibition.

In the other hand, G2-S24P dendrimer report similar behaviour but with lower HCMV_AD−169_ inhibition rate than G2-S16, in this case, at 24 h post infection G2-S24P dendrimer reaches 60% inhibition and maintains this activity for 72 h, at this time point the activity is similar to ganciclovir inhibition rate. Surprisingly, the combination of G2-S24P and ganciclovir shows an increased inhibition, reaching 90–95% for the first 48 h post infection, and decreases to 70–80% at 72 h and 96 h.

This increased inhibition rate is probably due to the combination of different mechanisms of action. Ganciclovir is a well know synthetic nucleoside analogue of guanine, related to acyclovir but has greater activity against HCMV and acts as DNA polymerase inhibitor [27].

In this respect, results on attachment, virucidal activity and time of addition, that compares G2-S16 and G2-S24P dendrimers with ganciclovir behaviour, may set an approximate mechanism of action of these dendrimers. However specific mechanism of action against HCMV_AD−169_ has not been deciphered yet. Both dendrimers show activity against HCMV_AD−169_ infection. We hypothesize, based on MTT assays (Fig. 2), that G2-S16 and G2-S24P dendrimers decrease mitochondrial activity, either by direct inhibition of mitochondria without causing global cell toxicity, or modifying other pathways in cell metabolism that may be covering mitochondrial activity in the MTT results. This reduction in mitochondrial activity is probably one of the key factors that induces the reduction of HCMV_AD−169_ infection, as this virus is known to depend on this pathway for production and assembly of viral proteins [28, 29].

## Conclusions

Summing up, treatments against HCMV infection started to fail in last years, as new resistances appeared. In addition, common treatments as ganciclovir or valganciclovir are not available in developing countries such as African continent, due to their high cost and no accessibility of HCMV tests. Thereby, the spread of HCMV infection in those countries is not reduced. Due to this facts, antiviral dendrimers could be a new approach in several fields. Its main role would be as a treatment or prophylaxis in countries with little accessibility to commercial antivirals, but also as a result of the good biocompatibility showed, it could be used in the field of transplant recipients. Dendrimer could eliminate the virus before the organ reach final host, or even so in congenital infections as HCMV is very present in vertical transmission. In either case G2-S16 or G2-S24P are presented as potent tools against HCMV either alone or in combination with the actual treatments. However, further studies should be performed to decipher the specific mechanism of action to develop new compounds for specific targets, for example in order to stop virus from crossing the placenta or to eliminate it in blood transfusions.

## Supplementary Information

**Additional file 1.** Nuclear Magnetic Resonance spectra of G1-S4 (DMSO), G2-S16 (D_2_O) and G2-S24P (D_2_O).

## Data Availability

The datasets used and/or analysed during the current study are available from the corresponding author on reasonable request.
