# Supplementary material for: Nanotechnology against human cytomegalovirus in vitro: polyanionic carbosilane dendrimers as antiviral agents
Source: J Nanobiotechnology. 2021 Mar 3;19:65. doi: 10.1186/s12951-021-00809-4 (PMC7927225; doi:10.1186/s12951-021-00809-4)
Supplement: Supplementary file 1 — Additional file 1. Nuclear Magnetic Resonance spectra of G1-S4 (DMSO), G2-S16 (D2O) and G2-S24P (D2O). [file 12951_2021_809_MOESM1_ESM.pdf]

Figure S1

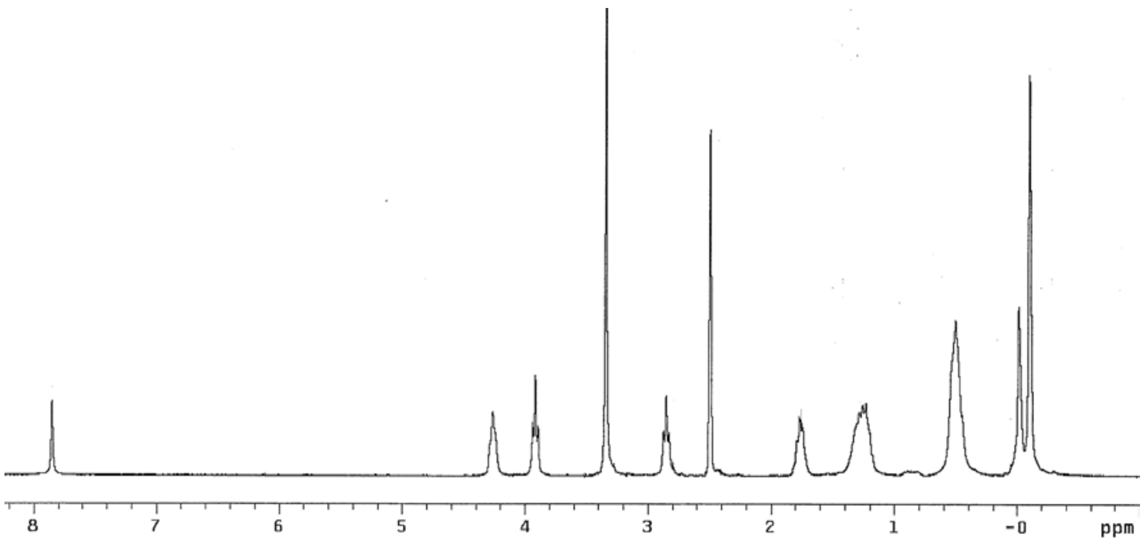

Figure S1. G1-S4 dendrimer with silicon core and 4 sulfate end groups was diluted in DMSO and analyzed by NMR.

Figure S2

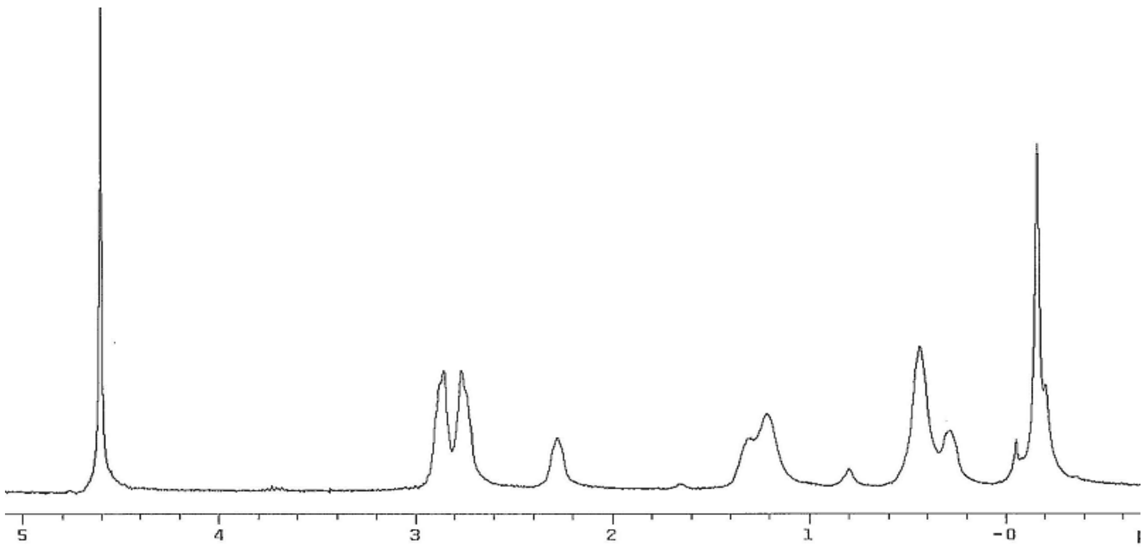

Figure S2. G2-S16 dendrimer with silicon core and 16 sulfonate end groups was diluted in water and analyzed by NMR.

Figure S3

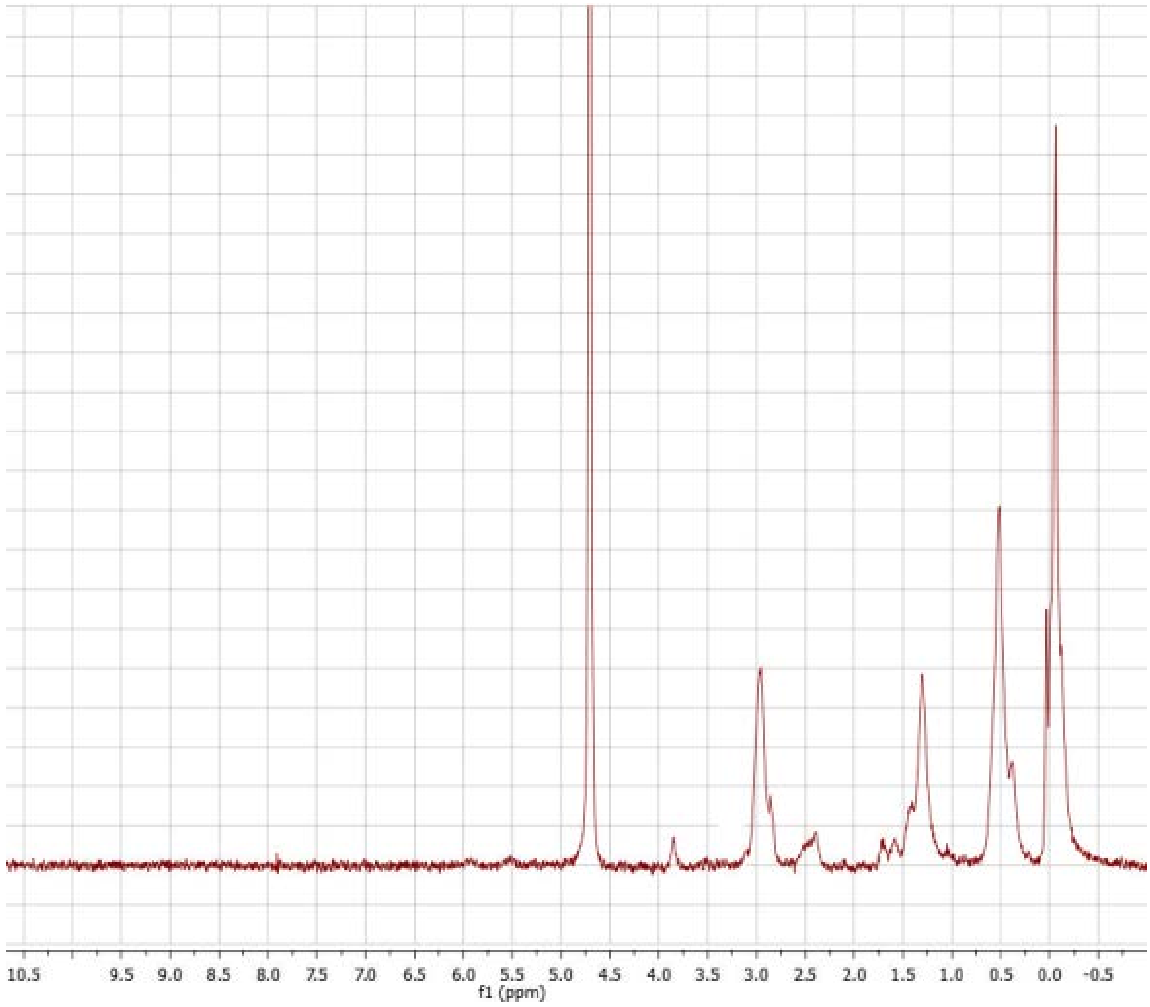

Figure S3. G2-S24P dendrimer with polyphenolic core and 24 sulfonate end groups was diluted in water and analyzed by NMR.
